# Autoimmune encephalitis associated with antibodies against intracellular antigens in children

**DOI:** 10.3389/fimmu.2025.1678497

**Published:** 2026-01-16

**Authors:** Linlin Zhang, Chao Che, Xingyu Han, Aihua Cao

**Affiliations:** 1 Department of Pediatrics, Qilu Hospital of Shandong University, Jinan, Shandong, China; 2 Department of Pediatrics, Qilu Hospital of Shandong University Dezhou Hospital, Dezhou, Shandong, China

**Keywords:** autoantibodies, autoimmune encephalitis, intracellular antigens, neurological syndromes, paraneoplastic

## Abstract

**Background:**

In neurological disorders associated with autoantibodies against intracellular antigens, response to therapy tends to be poor and is associated with irreversible neuronal death. This study aimed to analyze autoimmune encephalitis (AE) associated with antibodies (Abs) against intracellular antigens in children, clarify the clinical characteristics of the disease.

**Methods:**

We retrospectively analyzed patients with Abs against intracellular antigens at Qilu Hospital of Shandong University from 2024 to 2025. Detailed clinical characteristics were collected based on AE-Abs test results.

**Results:**

Fourteen pediatric patients with AE caused by intracellular Abs were recruited. The Abs included PCA-2, AGO, GFAP, SOX1, Ri, Yo, KLHL11, and AK5. All the patients received immunotherapy. After treatment, the antibody titers in the cerebrospinal fluid and serum of patients mostly decreased or even turned negative. Some patients were discharged with sequelae.

**Conclusion:**

In AE associated with Abs against intracellular antigens of children, seizures were the most common clinical manifestation. For patients with seizures without obvious inducement or suspected encephalitis, AE-related Abs should be detected promptly, and immunosuppressive therapy should be administered as early as possible. The prognosis of pediatric patients with encephalitis with Abs differs from that in adults, and most patients recover well after active treatment.

## 1 Introduction

Encephalitis is a severe inflammatory disorder of the brain with many possible causes and a complex differential diagnosis. Over the past decade, autoimmune encephalitis (AE) has experienced advances in identification of new syndromes and biomarkers. Patients with AE often present with memory or behavioral deficits without fever or alteration in the level of consciousness, or with normal brain magnetic resonance imaging (MRI) or cerebrospinal fluid (CSF) results (1). The laboratory hallmark of AE is bind to cell-surface or neural intracellular antigens represented by immunoglobulin G (IgG) autoantibodies (2).

AE antibodies (Abs) can be divided into two categories: autoantibodies, which target neuronal cell surface antigens, and intracellular antigens. Among pediatric AE, the former is dominated by the anti-N-methyl-D-aspartate, glycine, and gamma-aminobutyric acid type A receptors (3). In encephalitis associated with Abs against cell surface antigens, the Abs have access to the epitopes and can potentially change the structure and function of the cognate antigen (4). The latter mainly include Hu, Ma2, and glutamic acid decarboxylase (3). Abs that target neuronal epitopes (the target antigens are nuclear or cytoplasmic proteins, such as Yo, Hu, and Ma2) were first recognized in patients with paraneoplastic neuronopathy, encephalitis, or cerebellar degeneration (5). However, research has found that Abs to intracellular antigens cannot reach the intracellular epitopes, may not to be directly pathogenic, but rather are markers of T-cell responses that target neurons (5). In neurological disorders associated with these autoantibodies, response to therapy tends to be poor, which is associated with irreversible neuronal death (5).In recent years, with the advancement in antibody detection technology, an increasing number of Abs against the cell surface or intracellular antigens have been discovered. In adults, AE is commonly paraneoplastic, which is accompanied by the presence of an occult tumor that can act as a stimulus for autoantibody production (3). In childhood, non-paraneoplastic AE is more commonly diagnosed, however, the occurrence of paraneoplastic encephalitis with intracellular Abs is relatively less (3, 6). Therefore, this paper discusses 14 cases of children with AE associated with Abs against intracellular antigens; eight Abs targeting intracellular antigens were detected, of which five were classic and novel Abs with strong (>70%) oncological association (Yo; Ri; purkinje cell cytoplasmic antibody-2, PCA-2; SRY-related box-1, SOX1; Kelch-like protein 11, KLHL11), and one was a neural-specific Abs with moderate to low paraneoplastic associations (glial fibrillary acidic protein, GFAP). These Abs are relatively rare in children and are directed against intracellular antigens. We analyzed their clinical characteristics, CSF and imaging features, treatments, and prognosis, and provided new ideas for the diagnosis and treatment of this type of encephalitis.

## 2 Materials and methods

### 2.1 Standard protocol approvals, registrations and patient consents

The study was approved by the Ethics Committee of the Qilu Hospital of Shandong University. All patients or patients’ carers gave consent for the use of medical data for research.

### 2.2 Study design and participants

We conducted a retrospective, observational study with an analysis of relevant clinical information from patients who were associated with Abs against intracellular antigens in Qilu Hospital of Shandong University from 2024 to 2025. The inclusion criteria were as follows: age < 18 years, consistent with the diagnosis of paraneoplastic neurological syndromes (PNS) and AE, provisional positivity for Abs against intracellular antigens in the serum and/or CSF, and availability of clinical data, including demographic features, clinical manifestations, laboratory investigations, and brain MRI scans. The diagnosis of AE was based on the 2016 clinical criteria (1). For the diagnosis of PNS, we applied the 2021 Updated Diagnostic Criteria, utilizing the PNS-Care Score which evaluates the coherence between clinical phenotype, antibody risk category (High/Intermediate/Low), and the presence of malignancy (7).

### 2.3 Antibody detection

Samples from each patient in our hospital were sent to Guangzhou KingMed Diagnostics Group Co., Ltd for testing. A two-step screening strategy was employed. First, samples were screened using a Tissue-Based Assay (TBA) on composite rat brain tissue (hippocampus and cerebellum) to detect non-selective neuronal reactivity. To maximize sensitivity and specificity for high-risk intracellular and synaptic antigens, results were verified using fixed Cell-Based Assays (CBA) utilizing commercial transfected HEK293 assays. Positive results were evaluated at least in duplicate on the samples. Two independent masked assessors classified each sample as positive or negative based on the intensity of immunofluorescence in direct comparison with non-transfected cells and control samples. After positive samples were confirmed, the titer was determined by serially diluting them from 1:10 to 1:1000. The final titer was defined as the dilution value of the sample at which a specific fluorescence could just be recognized and expressed as the corresponding dilution value.

## 3 Results

### 3.1 Basic descriptive information

**Figure 1:**
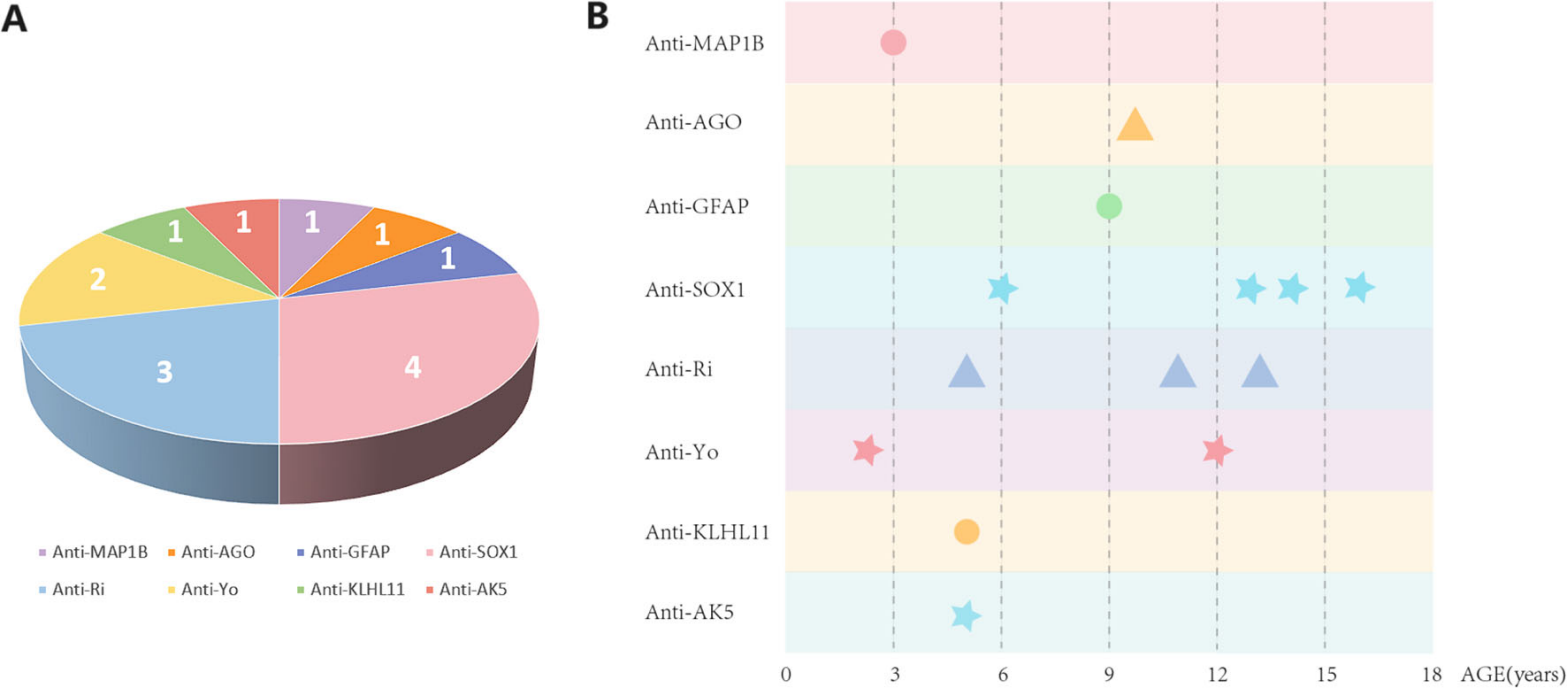
**(A)** The profile of the included patients with antibody-mediated autoimmune encephalitis. **(B)** Types of antibodies and the age distribution of pediatric patients.

In this investigation, data from 14 pediatric patients with AE caused by intracellular Abs were collected. The details are presented in **Table 1**. These included Abs against PCA-2 (coexisting with mGluR8), argonaute (AGO), GFAP, SOX1, Ri, Yo, KLHL11, and adenylate kinase 5 (AK5) (**Figure 1**). Of the 14 patients, six were male and eight were female. The oldest patient was 16 years old, and the youngest was only 2 years old. The onset was more concentrated in the age ranges of 3–6 years (4/14) and 12–15 years (4/14).

**Table 1:** Clinical features of children with antibodies against intracellular antigens.

No.	Age at onset	Sex	Clinical syndrome	Oncological comorbidity	EDSS score at onset	Antibodies	Treatment	Antibodies after treatment	EDSS score at followup
1	3y	F	Fever, vomiting, and repeated seizures	None	1.5	MAP1B Abs 1:30, mGluR8 Abs 1:10 and PCA-2 Abs 1:10 in serum detected by TBA	Steroids + IVIG + Meropenem and Cefmetazole	PCA-2 Abs (-) in serum	0
2	10y	F	Headaches, repeated seizures, joint pain and weakness in limbs	None	1.5	AGO Abs 1:10 in CSF and 1:30 in serum detected by CBA, positive in CSF and serum detected by TBA	Steroids + IVIG	AGO Abs (-) in both CSF and serum	1.0
3	9y	F	Fever, headaches, repeated seizures, double vision and weakness in limbs	None	2.0	GFAP Abs 1:32 in CSF and serum detected by CBA, positive in CSF and serum detected by TBA	Steroids +IVIG + PLEX + Meropenem and Vancomycin	GFAP Abs (-) in serum	1.0
4	16y	M	Fever, headaches, repeated seizures, lower limb joints and back pain	None	2.5	SOX1 Abs 1:30 in serum detected by CBA, positive in CSF and serum detected by TBA	Steroids + IVIG + Meropenem	No report	2.0
5	6y	M	Fever, vomiting, and seizures	None	2.0	SOX1 Abs 1:30 in serum detected by CBA, positive in serum detected by TBA	Steroids + Cefmetazole	No report	0
6	13y	F	Fever, chest pain, dyspnea	None	1.0	SOX1 Abs 1:30 in serum detected by CBA	Steroids + IVIG	No report	0
7	14y	M	Fever, headaches, and weakness in limbs	None	7.0	SOX1 Abs 1:30 in serum detected by CBA	Steroids + IVIG + PLEX + Meropenem	SOX1 Abs (-) in serum	5.0
8	13y	F	Fever, headaches, vomiting	None	1.5	Ri Abs 1:30 in serum detected by CBA	Steroids +IVIG + Cefoperazone Sulbactam and Voriconazole	Ri Abs 1:30 in serum	0
9	11y	M	Repeated seizures, fever	None	1.5	Ri Abs 1:100 in serum detected by CBA	Steroids +IVIG + PLEX + Lacosamine	Ri Abs 1:30 in serum	0
10	5y	M	Fever, unconsciousness and repeated seizures	None	1.5	Ri Abs 1:30 in serum detected by CBA	Steroids + IVIG + Meropenem + Ceftriaxone Sodium	Ri Abs 1:10 in serum detected by CBA	1.0
11	12y	F	Headaches, vomiting, repeated seizures and weakness in limbs	None	2.0	Yo Abs 1:100 in serum detected by CBA	Steroids +IVIG + Ganciclovir	No report	0
12	2y	M	Repeated seizures	None	2.0	Yo Abs 1:30 in serum detected by CBA	Steroids +IVIG + Ceftriaxone and Lacoxamide	No report	1.0
13	5y	F	Weakness and pain in both lower extremities, rash	None	3.0	KLHL11 Abs 1:30 in serum detected by CBA	Steroids + IVIG + Cefmetazole	No report	2.0
14	5y	F	Repeated seizures, unconsciousness	None	2.0	AK5 Abs 1:30 in serum detected by CBA	Steroids + IVIG + Euthyrox	AK5 Abs (-) in serum	2.0

y, year; F, female; M, male; EDSS, expanded disability status scale; MRI, magnetic resonance imaging; CSF, cerebrospinal fluid; OCB, oligoclonal bands; CBA, cell-based assays; TBA, tissue-based assays; PCA-2, Purkinje cell cytoplasmic antibody-2; AGO, Argonaute; GFAP, glial fibrillary acidic protein; SOX1, SRY-related box-1; KLHL11, Kelch-like protein 11; AK5, adenylate kinase 5; Abs, antibodies; IVIG, intravenous immunoglobulin; PLEX, plasma exchange.

### 3.2 Antibody detection and quantification

**Figure 2:**
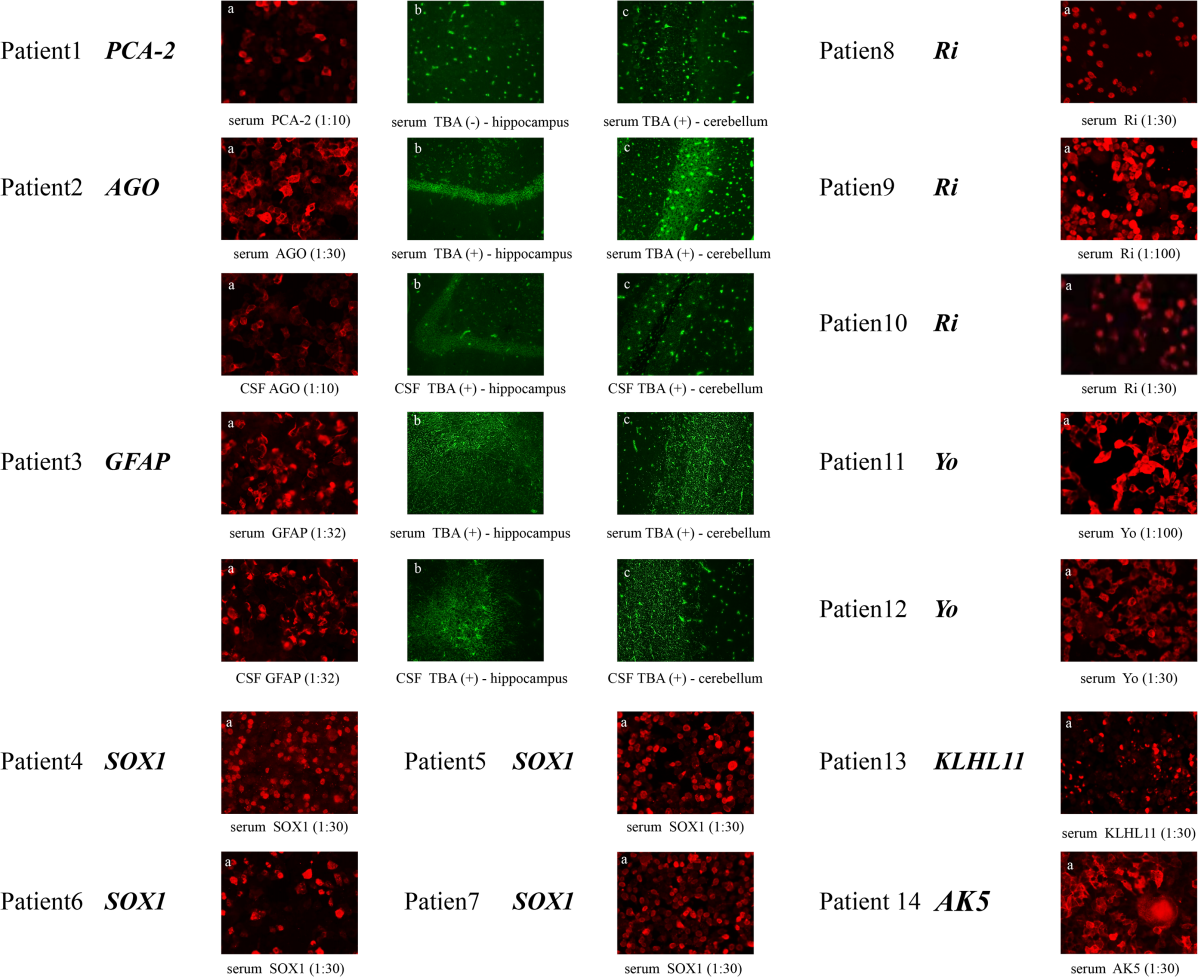
The legend shows the antibody detection results of the serum and CSF of the patients.

In our study, serum samples showed suspicious positive results for MAP1B Abs detected by TBA, which were verified by CBA to be weakly positive, with a titer of 1:30. CBA also revealed the coexistence of PCA-2 Ab (1:10) and mGluR8 Ab (1:10) in the patient’s serum. Anti-AGO and GFAP Abs were positive in both serum and CSF detected by TBA. AGO Abs were weakly positive in the serum and extremely weakly positive in the CSF, as detected by CBA, with titers of 1:30 and 1:10, respectively. According to the CBA measurements, the titer of GFAP Abs in the serum and CSF was 1:32, indicating weak positivity. As for SOX1 Abs, the CSF and serum Abs in one case were weakly positive with a titer of 1:30 detected by CBA; another was confirmed with a weak positive signature (1:30), while the remaining two cases were weakly positive for serum Ab titers of 1:30. Three serum samples were anti-Ri Ab positive, with titers of 1:30, 1:30, and 1:100. Positive anti-Yo Abs (1:30 and 1:100) were detected in the serum by CBA. One patient had a 1:30 positive KLHL11 Ab in the serum, quantified by CBA. The patient’s serum AK5 Ab level was 1:30 positive, as determined by CBA (**Figure 2**).

### 3.3 Clinical features

**Figure 3:**
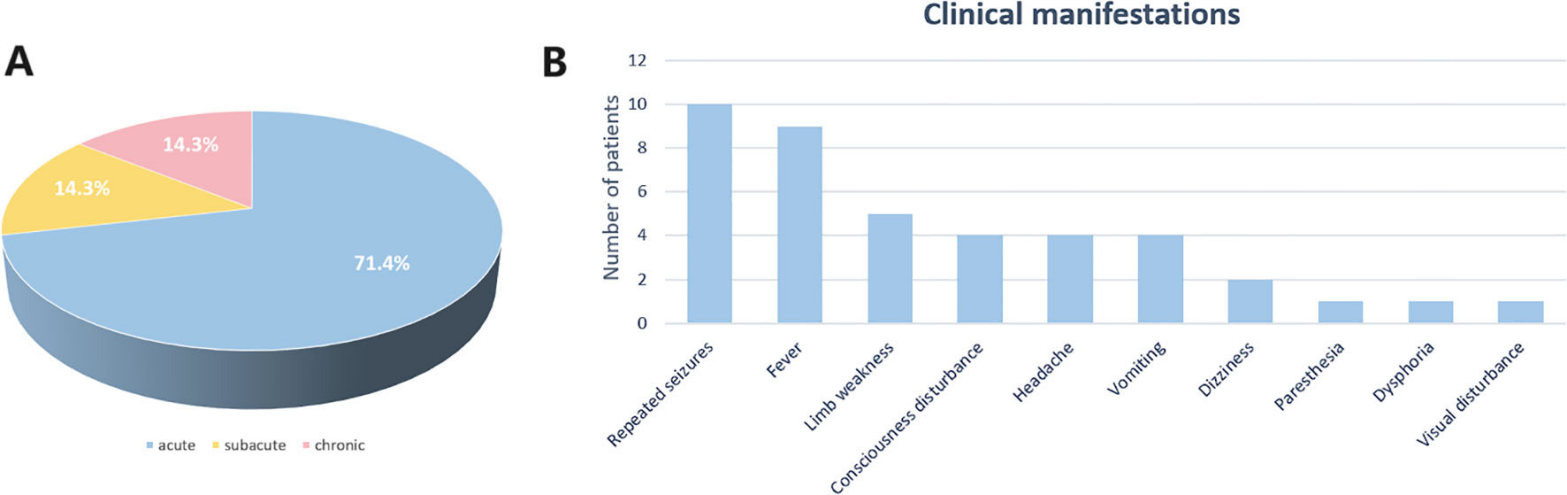
**(A)** Patient onsets of illness. **(B)** Patient clinical manifestations of illness.

Of the 14 included patients, 10 (71.4%) had acute onset (≤2 weeks), two (14.3%) had subacute onset (2 weeks-1 month), and two (14.3%) had chronic onset (≥1 month), as **Figure 3** illustrates. Among them, repeated seizures (10/14) and fever (9/14) were the most common clinical manifestations. Other accompanying symptoms included dizziness, paresthesia, and psycho-behavioral abnormalities such as dysphoria and visual disturbance. Except for one positive AK5-Ab case, all other types of Ab-positive cases had at least one patient presenting with repeated seizures. Individual patients who had positive anti-MAP1B or anti-GFAP manifested fever. Four patients positive for SOX1 Ab and three patients with anti-Ri Ab all suffered intermittent fever. Limb weakness was observed in children who tested positive for anti-AGO, anti-GFAP, anti-Yo (1/2), and anti-SOX1 (1/4) Abs. Dysphoria and visual disturbances were observed in patients positive for anti-GFAP Abs.

### 3.4 Auxiliary examinations

CSF examination was performed in all 14 patients. Two patients had leukocytosis (leukocyte count > 5 cells/mm3) with anti-GFAP and anti-SOX1 Abs; the highest recorded leukocyte count was 256 cells/mm3. In terms of biochemical results, different Abs presented with different alterations. Oligoclonal bands (OCB) were detected and a positive result was obtained only in patients with AK5 Ab. Only one patient had an abnormal manifestation of A. fumigatus detected by pathogen detection in the CSF. Other indicators that could be used to assess brain damage, such as neuron-specific enolase and S100 protein, were also tested. These two measurements were elevated in most patients who underwent the test compared to immunoglobulins, as shown in **Table 2**.

**Table 2:** Results of cerebrospinal fluid and brain injury related tests.

No.	Antibody	Biochemical examination			White blood cell	OCB	Pathogen detection	CSF immunoglobulin			Brain injury series (Serum detection)	
		Glucose	Chloride	Protein				IgG	IgA	IgM	Neuronspecific enolase	S100 protein
1	PCA-2	3	124	0.09↓	1	(-)	None	5.65	<1.03	<0.177	18.9↑	0.110↑
2	AGO	3.41	124	0.14↓	1	(-)	None	6.91	<1.03	<0.177	22.9↑	0.09
3	GFAP	2.69↓	127	0.46↑	52	(-)	None	85.3↑	9.5↑	1.41↑	38↑	4.770↑
		3.45	122	0.11↓	62	(-)	—	11.4	2.55	0.192	34.3↑	0.09
4	SOX1	2.48↓	111↓	1.10↑	256	(-)	None	93.9↑	12.2↑	5.090↑	39.2↑	0.910↑
		3.36	130	0.45	32	(-)	—	48.6↑	4.03	0.863	16.7↑	0.08
5	SOX1	4.15	125	0.13↓	1	(-)	None	7.2	<1.03	<0.177	22.10↑	0.08
6	SOX1	3.58	130	0.14↓	4	(-)	None	13.3	<1.14	<0.159	—	—
7	SOX1	4.91↑	123	0.2	1	(-)	None	17.6	1.39	<0.177	18.3↑	0.06
8	Ri	3.75	127	0.26	4	(-)	Aspergillus Fumigatus	17.3	2.17	0.214	—	—
		4.66↑	131↑	0.21	1	(-)	—	32.2	2.01	0.19	—	—
9	Ri	4.4	123	0.19	1	(-)	None	10.2	<1.03	<0.177	37.8↑	0.510↑
10	Ri-	4.3	127	0.16	12	(-)	None	11.6	1.35	1.620↑	—	—
11	Yo	3.7	127	0.19	4	(-)	None	14.4	1.2	<0.177	—	—
12	Yo	4.56↑	128	0.08↓	1	(-)	None	3.97	<1.03	<0.177	45.4↑	0.120↑
13	KLHL11	4.03↑	131↑	0.15	1	(-)	None	17.1	1.73	0.344	—	—
14	AK5	3.45	127	0.15	1	(+)	None	12.7	2	0.495	23.2↑	0.08

“—” indicates that the patient did not undergo the test. “↑” represents an increased level while “↓” represents a decreased level. OCB, oligoclonal bands; CSF, cerebrospinal fluid.

**Figure 4:**
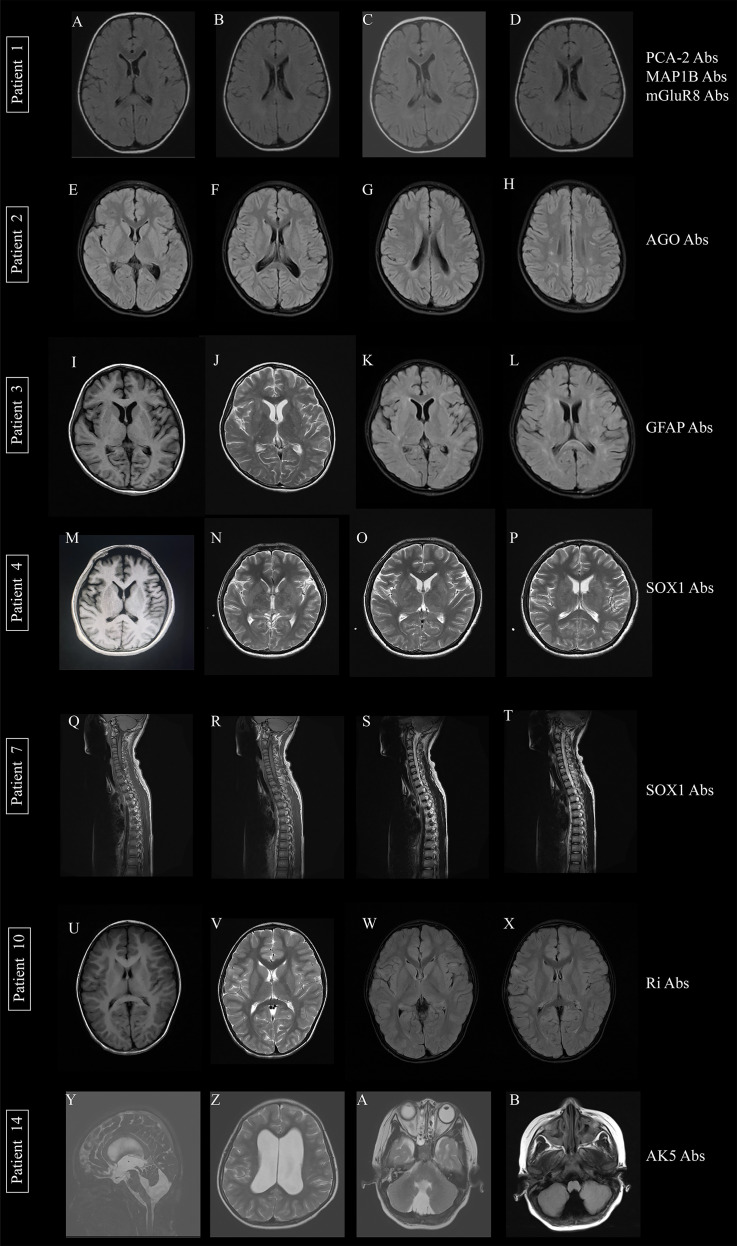
Brain MRI changes in patients with autoimmune encephalitis associated with antibodies against intracellular antigens. **(A, B)** Patient 1 with MAP1B Abs, mGluR8 Abs, and PCA-2 Abs, before treatment; **(C, D)** patient 1, after treatment; **(E–H)** patient 2 with AGO Abs, abnormalities around the bilateral radiative coronal and lateral ventricle triangle areas; **(I–L)** patient 3 with GFAP Abs, on T1WI, T2WI, and T2 FLAIR respectively; one of patients with SOX1 Abs, patient 4, showed abnormalities in the bilateral basal ganglia thalamus **(M–P)**, while another showed myelitis possibility on T1WI and T2WI **(Q–T)**; **(U–X)** the patient with Ri Abs showed long T1, T2 and T2 FLAIR signals; and (**Y, Z,** patient 14 **A, B**) the patient with AK5 Abs showed enlarged ventricle system.

All included patients underwent brain MRI. The distribution of lesions is shown in **Table 1** and **Figure 4**. In total, six patients had varying degrees of abnormal signals on MRI. Patients with the MAP1B Ab showed abnormal signals in the pressure region of the corpus callosum. In patients with positive AGO Abs, multiple abnormal signals of white matter in the brain were observed, and the possibility of pericerebral leukomalacia was considered. Acute disseminated encephalomyelitis was diagnosed in patients with GFAP Abs when combined with several aberrant signals on brain MRI and anamnesis. Two of the four anti-SOX1 Ab-positive patients showed no obvious abnormalities, while the other two had unusual signs in bilateral basal ganglia thalamic region and in the spinal cord from the submedulla to T1 level, respectively. Among the patients treated with anti-Ri Ab, only one showed multiple abnormal signals in the bilateral frontal lobe and cingulate gyrus, whereas two had no visible abnormalities. Patients with anti-Yo, anti- KLHL11, and anti-AK5 Abs displayed enlargement of the ventricles or lacuna, but no discernible changes in brain signals (two cases).

### 3.5 Treatment and prognosis

Immunotherapy included first-line (intravenous methylprednisolone (IVMP), intravenous immunoglobulin (IVIG), and plasma exchange (PLEX)) and second-line (rituximab, cyclophosphamide, and azathioprine) therapies. The different immunotherapies used in each patient with Ab-mediated central nervous system autoimmune diseases are shown in **Table 1**. All patients with encephalitis received first-line immunotherapy, and nine concurrently received antibiotic therapy. Lacosamide was also administered to patients 4, 5, 9, and 12 because of uncontrollable recurrent convulsions. Another Ri Abs-positive patient 8 with Aspergillus fumigatus existing in the CSF was administered voriconazole. Based on the biochemistry and cytology of the CSF of patient 10, which consisted mainly of lymphocytes, viral encephalitis was considered, and acyclovir antiviral therapy was administered. Notably, nucleic acid tests for pathogens in the CSF of patient 11 with anti-Yo Ab were positive for human herpesvirus type 6 (HHV-6); therefore, ganciclovir was administered concurrently to antiviral.

After treatment, the Ab titer of the CSF and serum of patients mostly decreased or even turned negative (**Table 1**). However, Ri Abs did not completely turn negative after treatment. The titer in one of the three patients was 1:30 before and after treatment, although the symptoms improved. In the other two cases, the titer decreased from strongly positive 1:100 to weakly positive 1:30, and from 1:30 to extremely weak 1:10. Patient 2 tested negative for anti-AGO Abs after treatment, however, the patient still had mental abnormalities and continued treatment in a rehabilitation hospital after discharge. The weakness of the limbs in patient 3 gradually improved after treatment, and there were no frequent convulsions. However, treatment was continued in the rehabilitation department because of difficulties with spontaneous urination. Patient 7 experienced limb weakness on admission. After treatment, the patient was discharged with grade 2 proximal muscle strength in both upper limbs and grade 0 muscle strength in both lower limbs, and continued rehabilitation exercises outside the hospital. Physical examination of patient 13 showed weakness in both lower limbs upon admission. After treatment, her muscle strength improved with both lower-limb and upper-limb muscle strength IV. Some of these patients underwent re-examination with brain MRI, as shown in **Table 3**. Most of them showed better imaging results than before or even no abnormal findings.

**Table 3:** Detailed MRI results.

No.	Antibody	Manifestation	Reexamination
1	PCA-2	Long T1 and long T2 signals in the pressure part of the corpus callosum, manifested as high signals in DWI and low signals in ADC. Reversible lesions possibility	No obvious abnormal signals in the brain parenchyma.
2	AGO	Multiple elongated T1 and T2 signals around bilateral radiative coronal area and lateral ventricle triangle area. High signals in T2 FLAIR. Equal signal in DWI. Periventricular leukomalacia possibility	—
3	GFAP	Flaky long T1 and long T2 signals in the pressure part of the corpus callosum, and T2 FLAIR showed high signals. At T1–10 level, high signals on T2WI in the anterior part of the spinal cord. Acute disseminated meningomyelitis possibility	Slightly reduced degree and range of the signalcompared with the pre-signal
4	SOX1	High signals in lamellar FS-T2WI and superficial fasciitis possibility. Long T2 signals in the bilateral basal ganglia thalamus, high signals in T2 FLAIR with radially distributed along the perivascular space in sagittal position. The possibility of inflammatory changes	In the thalamic area of the basal ganglia, brain stem and bilateral pontine arms, point-like long T2 signal and high T2FLAIR signals
5	SOX1	No abnormal signals in the brain parenchyma	—
6	SOX1	Small cystic signal shadow in pineal region with 7mm in diameter	—
7	SOX1	No abnormal signal shadow in the brain parenchyma. The spinal cord thickened from the submedulla oblongata to the T1 level, and high signal shadow on lamellar T2WI. Myelitis was considered	Straight cervical spine and reduced scope of the patchy, longer T2 signal in the cervical pulp at the level of C2-6
8	Ri	No abnormal signals in the brain parenchyma	—
9	Ri	No abnormal signals in the brain parenchyma	—
10	Ri	Multiple flecks of long T1, long T2, and T2 FLAIR in the bilateral frontal lobe, cingulate cortex and subcortex, with slightly high DWI signals. The possibility of encephalitis	Performance as before, slightly wider range
11	Yo	No abnormal signals in the brain parenchyma	—
12	Yo	No abnormal signals in the brain parenchyma	—
13	KLHL11	No abnormal signals in the brain parenchyma	—
14	AK5	Punctate T2 signals were seen in the aqueduct area of the midbrain. Cerebrospinal fluid film showed enlarged ventricle system, enlarged posterior cistern and cisterna magna, and a slightly smaller inferior worm of the cerebellum	—

## 4 Discussion

In our study on AE associated with Abs against intracellular antigens in children, repeated seizures, fever, limb weakness, consciousness disturbance, headache, and vomiting were the most common clinical manifestations. Other accompanying symptoms included dizziness and paresthesia, while psycho-behavioral abnormalities such as dysphoria and visual disturbance were also observed. Of these 14 patients, 10 presented with repeated seizures. If previously healthy children present with new focal or diffuse neurologic deficits, cognitive impairment, developmental regression, movement abnormalities, psychiatric symptoms, and/or seizures in less than 3 months, a diagnosis of pediatric AE should be considered (8). In Ab-mediated encephalitis, seizures can be the first, the predominant, or the only manifestation of the disease (9). Seizures are extraordinarily frequent in the inflammatory-provoked phase, and would resolve after the encephalitis abates (9). However, the mechanism driving the immune response influences the susceptibility to causing enduring seizures in AE, with intracellular antigen-associated encephalitis mediated by cytotoxic T cells (CTLs) have high predisposition, and surface antigen-associated encephalitis mediated by Abs have moderate or absent predisposition (9). Clinically, seizures may be the sole manifestation of AE, a presentation often classified as New-Onset Refractory Status Epilepticus (NORSE). According to the 2022 International Consensus Recommendations for the Management of NORSE/FIRES, diagnostic workup should not delay treatment. In cases where structural, toxic, or metabolic causes are excluded, empiric first-line immunotherapy is recommended within 72 hours of seizure onset to prevent irreversible neuronal injury, even before antibody results are available (10). In all patients, 12 were associated with paraneoplastic Abs. PNS is a group of neurological disorders that are secondary to an immune response triggered by the underlying tumor, which affects the central or peripheral nervous system. Most classic paraneoplastic syndromes are associated with Abs against intracellular (onconeural) antigens, appear to be mediated by CTL responses, and have limited response to treatment (11).

There was one patient had two Abs combined in our study. PCA-2 IgG is a paraneoplastic biomarker and MAP1B protein is the autoantigen target for PCA-2-IgG (12, 13). The expression of MAP1B in the dorsal root ganglia and peripheral nerves support that MAP1B specific T-cells contributing to observed paraneoplastic neuropathy phenotype (13, 14). Clinical presentation associated with MAP1B-IgG is variable and often a subacute and progressive neurological syndrome (12, 13). Autonomic dysfunction or gastrointestinal dysmotility was present only in a subset of these patients (14). The case presented in this paper showed neurological symptoms of recurrent convulsions, as well as gastrointestinal dysmotility of intestinal obstruction, which were consistent with the clinical manifestations of MAP1B-IgG-related lesions. As is often the case for paraneoplastic neural Abs, MAP1B IgG commonly coexists with other neural Abs, and the frequency of its coexisting neuronal nuclear or cytoplasmic auto ab for is 50% (15). In our case, in addition to the positive MAP1B-IgG, we also detected mGluR8 Abs. The expression of mGluR8 in the gut and pancreas suggests that this receptor is involved in gastrointestinal motility and insulin secretion *in vivo* (16). mGluR8 Ab is present in this case, which is similar to MAP1B-IgG, and has effects on both the nervous system and gastrointestinal movement.

SOX1 is the antigen recognized by anti-glial nuclear Abs (AGNA)–positive sera (17). The neurological dysfunction associated with anti-SOX1 Abs may involve multiple levels of the neuroaxis, including the limbic system, cerebellum, peripheral nervous system, and neuromuscular junction (18). Similar to a previous study, the clinical manifestations were varying in children with anti-SOX1 Abs in our research; all patients had fever, followed by headache, vomiting, convulsions, and limb weakness. However, compared with adult patients in previous studies, the clinical manifestations of paraneoplastic Abs associated disease in children seem to be less severe, similar to other AE, and most of them have symptoms such as fever, headache, and repeated seizures. We also identified GFAP, and two Abs with no clear paraneoplastic associations, AK5 and AGO. Autoimmune GFAP astrocytopathy is a recently described autoimmune disease. Its clinical features are similar to meningoencephalomyelitis, encephalitis, meningitis, and myelitis (19). AGO Abs might be potential biomarkers of autoimmunity in patients with central and peripheral nonparaneoplastic neurologic diseases including sensory neuronopathies, LE and, less frequently, cerebellar syndrome, opsoclonus–myoclonus, and length-dependent polyneuropathies (20). AGO2 Abs have been reported in 10–20% of patients with various rheumatic diseases, including systemic lupus erythematosus, polymyositis/dermatomyositis and other rheumatologic autoimmune diseases (21). One patient in our study first presented with headache and intermittent convulsions, and was associated with joint pain, a small amount of effusion in knee MRI, and positive anti-Ro-52 Abs and anti-nuclear Abs; thus, it is necessary to be alert to the possibility of rheumatic diseases.

For patients with anti‐Yo Abs, MRI of the brain is often normal in the early stages, with cerebellar atrophy seen later (22). Although T2 signal alterations in the brainstem and cerebellum have been reported, an MRI of the brain may be mostly normal in patients with anti‐Ri Abs (23). However, the brain MRI of a patient in our research showed multiple abnormal signals in the bilateral frontal lobe and cingulate gyrus. In previous studies, 46.7% of the patients for anti-SOX1 Abs showed normal MRI findings (18). Abnormal cerebellar changes were observed in only three of the 30 patients with anti-SOX1 Abs, two patients with PCD had cerebellar atrophy, and one patient with PCD had diffuse hyperintense lesions in T2-weighted imaging of the cerebellum and brainstem and had bad marked atrophy 2 months into treatment (24, 25). Other abnormal MRI includes longitudinal T2 hyperintensity of the thoracal spinal cord and bilateral T2 hyperintensity temporo-mesial and hippocampus (24). In our study, two of the four patients had normal MRI findings. One patient’s brain MRI revealed abnormal signals in the bilateral basal ganglia, thalamus, brainstem, and pontine brachium. Another patient with limb weakness showed multiple abnormal signals in the spinal cord from the lower medulla oblongata to the T1 level, suggesting myelitis. The typical clinical presentation of paraneoplastic KLHL11 encephalitis is rhombencephalitis; the brain MRI is initially normal in a considerable minority of patients and often demonstrates T2/FLAIR abnormalities involving the brainstem or limbic system (26). In this study, no abnormalities were found on brain MRI; however, abnormal signals were found on MRI of the lower limb muscles, which were considered to have myositis or myopathy. In GFAP astrocytopathy, characteristic imaging findings known as paraventricular linear radial enhancement were revealed (27). Brain MRI in our study showed multiple abnormal signals in the corpus callosum and T2–10 spinal cord, and the possibility of acute disseminated encephalomyelitis was considered. Research showed that all patients had bilateral hippocampal hypersignal on brain MRI of patients with AK5 Abs (28). In our case, brain MRI of a child showed that the ventricle system was enlarged; however, considering that the growth and development of the child were in retardation, regular review and assessment of the condition were required.

In our case, the symptoms gradually improved after treatment with steroids and IVIG, but it is still necessary to evaluate the patient’s recovery and regularly review the ultrasound findings to rule out the possibility of a tumor. Most patients with PCA-2 showed good responses to immunotherapy and cancer treatment, and they often had favorable clinical outcomes (29). Similar to the study above, our patients recovered well after aggressive immunotherapy with no other sequelae. Studies have shown that most patients with GFAP have a favorable prognosis, while a few are poorly responsive to treatment and some may develop sequelae with varying degrees of functional disability (19). A previous study showed that despite receiving corticosteroids, IVIG, and PLEX, both of the two patients with AK5 Abs had relentless neurological deterioration and were poorly responsive to treatment; thus aggressive immunosuppressive therapy should be considered (30). One patient in our report was diagnosed with AK5 Abs and his condition improved after aggressive immunosuppressive therapy. A recent study found that AGO Ab production could be a marker of a remarkably severe inflammatory process, and prompt initiation of aggressive immunosuppressive treatment is of vital importance (31). Seizures in paraneoplastic encephalitis or pain in paraneoplastic neuropathies may show early improvement within 4–6 weeks of initiating immunotherapy. However, cognitive impairment and motor or sensory deficits usually recover more slowly (32). After treatment, the Ab titer of the CSF and serum of patients mostly decreased or even turned negative, and the clinical symptoms also improved. In summary, ten patients had recurrent convulsions during treatment but did not develop seizures again during follow-up. Some patients were discharged with sequelae such as mental abnormalities and limb dysfunction and required sequential rehabilitation treatment to alleviate the sequelae.

The present study has some limitations. Our study was mainly limited by a small and heterogeneous sample size. This study focused mainly on AE Abs and clinical data to analyze the findings, the lack of information on the follow-up of children makes it difficult to determine whether tumors and other diseases occur. Based on the results of the present study, Abs against intracellular antigens are mostly associated with paraneoplastic Abs. In addition to the common clinical manifestations of encephalitis, such as fever, headache, vomiting, and repeated seizures, limb weakness and disturbances in consciousness may also occur. Seizures were the most common clinical manifestation. In addition, due to the presence of Abs against intracellular antigens in all of our cases, the response to therapy is also relatively difficult. Therefore, for patients with seizures without obvious inducement and suspected encephalitis, AE-related Abs should be detected promptly using a comprehensive approach that includes TBA for broad screening, followed by CBA or immunoblotting for specific antigen confirmation, and immunosuppressive therapy should be performed as early as possible, which is very important for improving patient prognosis.

## Data Availability

The original contributions presented in the study are included in the article/supplementary material. Further inquiries can be directed to the corresponding author.
